# Percutaneous Continuous Radiofrequency Versus Pulsed Radiofrequency Thermorhizotomy for the Treatment of Neuralgia of the Trigeminal Nerve: A Retrospective Observational Study

**DOI:** 10.7759/cureus.54830

**Published:** 2024-02-24

**Authors:** Juan Acevedo-Gonzalez, Juan Jose Perez-Ocampo, Maria Alejandra Bautista-Piñeros, Sofia de los angeles Acosta-Rivas

**Affiliations:** 1 Neurosurgery, San Ignacio University Hospital, Pontifical Xavierian University, Bogota, COL; 2 Neurological Surgery, San Ignacio University Hospital, Bogota, COL; 3 Neurological Surgery, Pontifical Javierian University, Bogota, COL

**Keywords:** radiofrequency, trigeminal neuralgia, trigeminal, neuralgia, chronic pain, pain

## Abstract

Objectives: Trigeminal neuralgia (TN) represents one of the most powerful manifestations of neuropathic pain. The diagnostic criteria, as well as its therapeutic modalities, stand firmly established. The percutaneous radiofrequency thermorhizotomy of the gasserian ganglion and posterior root of the trigeminal nerve stands as a widely employed procedure in this context. In this retrospective observational investigation, we undertake a comparative analysis of patients subjected to treatment employing continuous radiofrequency (C-rF) versus pulsed radiofrequency (P-rF).

Materials and methods: A cohort of 128 patients afflicted with essential neuralgia of the trigeminal nerve, all under the care of the distinguished author (JCA), underwent percutaneous radiofrequency thermorhizotomy between the years 2005 and 2022. They were stratified into two cohorts: Group 1 encompassed 76 patients treated with C-rF, while Group 2 comprised 52 patients subjected to P-rF intervention. All participants met the stringent inclusion and exclusion criteria for TN, with a notable concentration in the V2 and V3 territories accounting for 60% and 45%, respectively. The post-procedural follow-up period exhibited uniformity, spanning from six months to 16 years. Preceding the intervention, all patients uniformly reported a visual analog scale (VAS) score surpassing 6/10. Additionally, everyone had been undergoing pharmacological management, involving a combination of antineuropathic agents and low-potency opioids.

Results: The evaluation of clinical improvement was conducted across three temporal domains: the immediate short-term (less than 30 days), the intermediate-term (less than one year), and the prolonged-term (exceeding one year). In the short term, a noteworthy alleviation of pain, surpassing the 50% threshold, was evident in most patients (94%), a similarity observed in both cohorts (98% in Group 1 and 90% in Group 2). The VAS revealed an average rating of 3/10 for Group 1 and 2/10 for Group 2. Moving to the intermediate term, more than 50% improvement in pain was registered in 89% of patients (92% in Group 1 and 86% in Group 2). The mean VAS score stood at 3.5/10, marginally higher in Group 2 at 4/10 compared to 3/10 in Group 1. In the final assessment, a 50% or greater reduction in pain was reported by 75% of patients, with no discernible disparity between the two cohorts. Among the cohort, 18 individuals necessitated a subsequent percutaneous intervention (10 in Group 1 and eight in Group 2), while microvascular decompression was performed on six patients (equitably distributed between the two groups), and radiosurgery was administered to three patients in Group 1.

Conclusions: Percutaneous radiofrequency thermorhizotomy emerges as an efficacious modality for addressing essential TN. The employment of continuous radiofrequency yields superior long-term outcomes, albeit accompanied by sensitive manifestations that may prove discomforting. Pulsed radiofrequency demonstrates commendable clinical efficacy with a diminished incidence of complications, rendering it a viable option even for other manifestations of facial pain.

## Introduction

Since antiquity, dating back to the erudition of ancient civilizations including the works of Galen and Aretaeus of Cappadocia, the formidable nature of facial pain has been underscored [1]. Avicenna of ancient Persia, perceiving it as "torture oris," was one of the pioneers to distinguish this neuropathic pain from other somatic afflictions [2]. In the annals of 1756, Nicolas André coined the term "tic douloureux," elucidating its paroxysmal aspect akin to convulsions. It was notably recognized as Fothergill's disease, an eponym stemming from the inaugural depiction of 14 patients afflicted with this ailment by the Edinburgh-born luminary, Fothergill, before the assembly of the London Medical Society [3]. In his perspective, contrary to André's notion, the etiology of these agonies lay in malignant conditions [3].

After the advent of pioneering antineuropathic agents in the 1950s, namely diphenylhydantoin and later carbamazepine, a multitude of ablative therapeutic modalities were contemplated [2]. These encompassed the application of agents such as alcohol and glycerol as well as mechanical and surgical interventions targeting both the peripheral nerve and the gasserian ganglion through the conduit elucidated by Hartel, employing radiofrequency waves [4].

The term "radiofrequency," more commonly recognized as the "radiofrequency spectrum," designates the segment of the extensive electromagnetic spectrum characterized by its comparatively lesser energy, spanning from 3 kHz to 300 GHz [5]. In 1906, the erudite German physician Karl Franz Nagelschmidt pioneered the concept of diathermy, which signifies the generation of heat within bodily tissues through the application of a high-frequency electrical current between two electrodes strategically positioned on the dermis [5]. Cushing propagated the utilization of diathermy and introduced the term "electrosurgery" to denote procedures employing radiofrequency waves for tissue coagulation and resection. In the realm of pain management, it was Dansorval who administered these waves to human tissues in 1891, thereby corroborating their capacity to elevate temperature upon contact [6-8].

The inaugural commercialized apparatus designed for employing radiofrequency in pain management was conceived in 1950 by Cosman and Aranow [9,10]. In 1995, Ayrapetyan et al. revolutionized the field by advancing the concept of pulsed radiofrequency, or isothermal radiofrequency, as a novel non-invasive and non-destructive therapeutic modality [11]. This variant of radiofrequency engenders electromagnetic fields that elicit physiological effects on cellular membranes, affecting alterations in their structural integrity. It induces thermal elevation, albeit at temperatures that remain non-deleterious, and interacts with the tissue through cycles of waves that permit the tissue to convalesce from the temperature surge occurring between cycles [12]. These breakthroughs were subsequently refined by Sluijter et al. [12].

In this study, we present a cohort of patients afflicted with essential trigeminal neuralgia (TN), all subjected to percutaneous radiofrequency thermorhizotomy. We meticulously scrutinize their demographic attributes and assess their outcomes in the immediate, intermediate, and protracted temporal domains. Furthermore, we conduct a comparative analysis between patients treated with continuous radiofrequency and those subjected to pulsed radiofrequency.

## Materials and methods

In a comprehensive retrospective analysis spanning from January 2005 to December 2022, a meticulous examination was performed on patients diagnosed with essential neuralgia of the trigeminal nerve who received treatment from one of the authors (JCA). The therapeutic intervention encompassed radiofrequency percutaneous thermorhizotomy, which precisely targeted the gasser ganglion and the posterior root of the trigeminal nerve. This endeavor entailed a thorough perusal of the patients' medical records, culminating in the creation of an exhaustive database comprising critical epidemiological facets including age, gender, disease progression timeline, pain localization, and the prescribed treatment regimen. Furthermore, fundamental procedural elements were scrutinized, and the clinical outcomes were appraised utilizing the VAS, with due consideration given to medication utilization and any attendant complications. The temporal trajectory of post-procedural monitoring, along with any exigencies for additional interventions to manage pain, was meticulously documented. The analytical endeavor was punctuated by the stratification of the patient cohort into two distinct groups: Group 1, comprising those subjected to continuous radiofrequency (C-rF), and Group 2, encompassing patients treated with pulsed radiofrequency (P-rF). Notably, rigorous adherence to inclusion and exclusion criteria was a paramount consideration throughout this investigative pursuit (Table 1).

**Table 1:** Inclusion and exclusion criteria

Inclusion criteria
Patients with a confirmed diagnosis of essential trigeminal neuralgia in accordance with globally recognized criteria
Prior cranial resonance imaging study
Antecedent multimodal pharmacological intervention
Exclusion criteria
Secondary trigeminal neuralgia
Postherpetic neuralgia
Diagnosis of multiple sclerosis or compression pathologies on the trigeminal nerve
History of treatment with radiosurgery
History of previous surgical treatment (microvascular decompression)
History of lytic procedures on the peripheral nerve

Patient demographics

A total of 128 individuals were under the care of the same specialist from January 2005 to December 2022, receiving radiofrequency percutaneous thermorhizotomy according to the Hartel technique. They were stratified into two distinct groups: Group 1, comprising 76 patients (59%) subjected to C-rF, and Group 2, consisting of 52 patients (41%) treated with P-rF.

Age distribution

The patients' ages spanned from 22 to 82 years, with an overall mean age of 57 years. Within Group 1, the average age was 72 years, demonstrating a range from 45 to 82 years. In contrast, Group 2 exhibited an average age of 42 years, encompassing an age range from 22 to 58 years.

Gender composition

Among the cohort, 89 individuals were female, accounting for 69% of the total, while 39 were male, constituting 28.9% of the population. Notably, in both Group 1 and Group 2, women were the predominant demographic, with respective ratios of 49 (64.4%) to 27 and 40 (76.9%) to 12.

Follow-up duration

Each patient received clinical oversight for a duration ranging from six months to 16 years, with an average follow-up period of six years. Importantly, there were no discernible disparities in follow-up duration between the two groups.

Clinical profile

All patients presented with essential neuralgia of the trigeminal nerve, meeting the meticulously defined inclusion and exclusion criteria. The duration of pain evolution exceeded 3.5 years in most cases, ranging from six months to 20 years in Group 1, and one to 15 years in Group 2. Pain manifested concurrently in the V2 and V3 territories for both Group 1 and Group 2, accounting for 60% (45 patients) and 45% (23 patients) of cases, respectively. Notably, pain localized solely in the V1 territory was observed in a modest 8% of cases (six patients) in Group 1 and 10% of cases (five patients) in Group 2. Preceding the procedure, all patients reported pain levels surpassing 6 on the VAS, with an average score of 8/10 in Group 1 and 9/10 in Group 2.

Prior interventions

At the time of the procedure, all patients were undergoing pharmacological management, encompassing antineuropathic and/or opioid regimens. Remarkably, over 70% (89 patients) were prescribed more than one medication, predominantly a combination of antineuropathic and opioid agents, with percentages of 78% (59 patients) in Group 1 and 80% (41 patients) in Group 2. Moreover, a notable fraction, exceeding 40% (51 patients), had previously undergone anesthetic infiltrations targeting the peripheral branches of the trigeminal nerve. This was observed in 38 patients (50%) from Group 1 and 20 patients (38.4%) from Group 2. Additionally, nearly half of the patients in both groups had received prior dental treatment, with or without associated dental extractions, accounting for 40% of cases (51 patients) (Table 2).

**Table 2:** Data summary of patients treated with continuous radiofrequency (Group 1) and pulsed radiofrequency (Group 2) VAS: Visual analog scale.

	Group 1: Continuous radiofrequency	Group 2: Pulsed radiofrequency	Total
Number of patients	76 (59%)	52 (41%)	128
Age	Mean age: 72 years; Range: 45-82 years	Mean age: 42 years; Range: 22-58 years	Mean age: 57 years; Range: 22-82 years
Sex	49 females/27 males	40 females/12 males	89 females (69%)/39 males (31%)
Time of clinical evolution	6 months-20 years	1-15 years	6 months-16 years
Topography of pain	V2 + V3 60% V1 8%	V2 + V3 45% V1 10%	
Previous treatment	78% pharmacological management (two or plus) 38 patients: peripheral block nerve	80% pharmacological management (two or plus) 20 patients: peripheral block nerve	70% pharmacological management; 40% peripheral nerve block; 40% dental extractions
Previous VAS	8/10 (average rating)	9/10 (average rating)	8.5/10 (average rating)
Short-Term VAS	3/10 (average rating) (98% of patients with more than 50% improvement)	2/10 (average rating) (90% of patients with more than 50% improvement)	94% of patients with more than 50% improvement
Intermediate-term VAS	3/10 (average rating) (92% of patients with more than 50% improvement)	4/10 (average rating) (86% of patients with more than 50% improvement)	3.5/10 (average rating) (89% of patients with more than 50% improvement)
Prolonged term (improvement more than 50%)	75%	75%	75%
Complications	Mortality: 0%; Morbidity: III cranial nerve palsy (1 patient), corneal hypoesthesia (8 patients), facial hypoesthesia (76 patients)	Mortality: 0%; Morbidity: facial hypoesthesia (6 patients)	
Other treatments	Subsequent percutaneous intervention (10 patients); radiosurgery (3 patients); microvascular decompression (3 patients)	Subsequent percutaneous intervention (8 patients); microvascular decompression (3 patients)	

Percutaneous procedure

All patients underwent the procedure under the expert care of the same specialist, JCA, within the confines of the surgical theater. Patients were positioned supine with their heads maintained in a neutral, slightly extended orientation. The application of fluoroscopy (C-arm) in a stringent lateral position ensured precise alignment of the sphenoid planum, the floor of the sella turcica, and the ascending ramus of the lower jaw. Identification of Hartel's pyramid points on the skin marked the initiation of the procedure. Subsequently, the surgical site was meticulously cleansed. Employing a predetermined anesthetic protocol, augmented by neurosedation, 1 cc of bupivacaine was judiciously injected at the puncture site.

**Figure 1:**
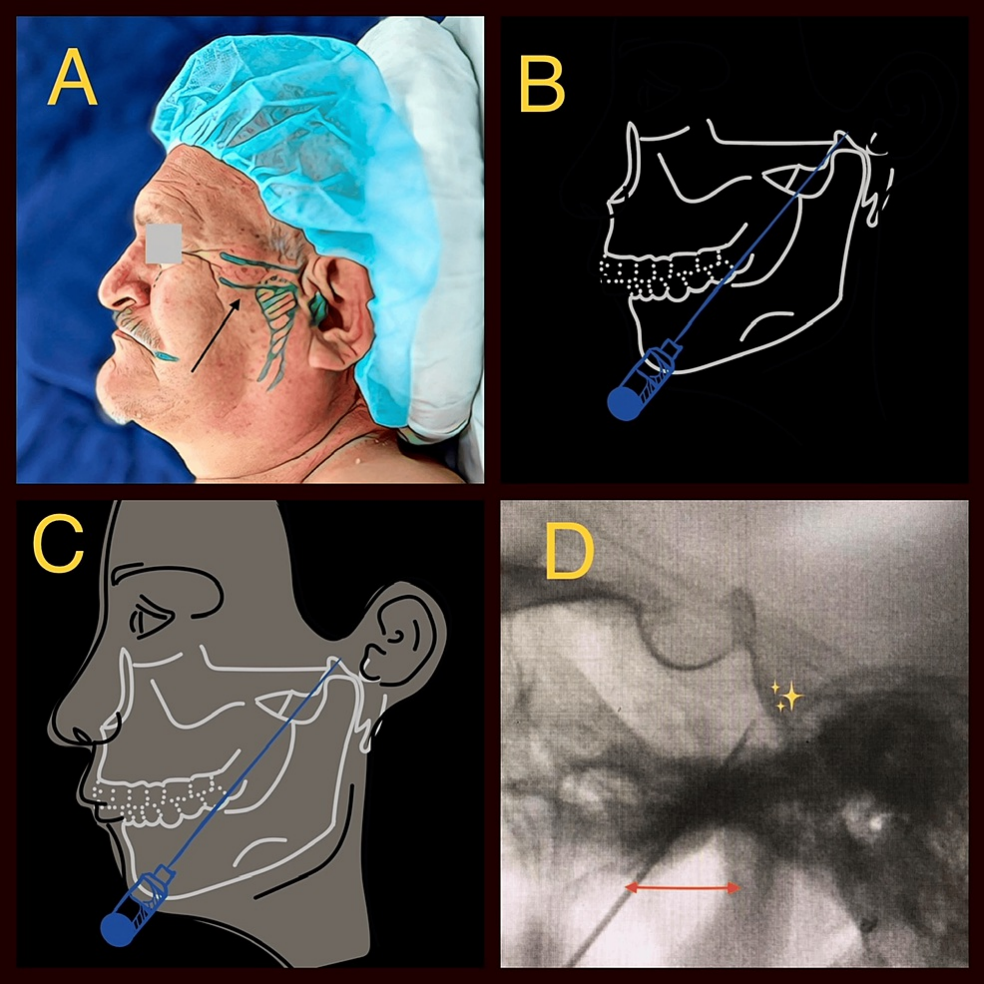
Percutaneous procedure (A) Patients were positioned supine with their heads maintained in a neutral, slightly extended orientation. The application of fluoroscopy (C-arm) in a stringent lateral position ensured precise alignment of the sphenoid planum, the floor of the sella turcica, and the ascending ramus of the lower jaw. (B and C) The puncture was executed with a 10 cm x 1 cm radiofrequency cannula, an area conspicuously devoid of Teflon. The operator's index finger was introduced into the patient's oral cavity, tracing along the upper jaw. A pivotal directive was to ensure that the needle never exceeded medial limits, safeguarding against inadvertent carotid puncture, as the foramen lacerum resides medially to the upper jaw's edge. (D) With the aid of fluoroscopy imagery, the needle was deftly guided along an imaginary projection line, passing 1 cm anterior to the temporomandibular joint (TMJ), a structural landmark impeccably discernible on strictly lateral radiographs. Image credit: This figure was created by one of the authors, Acevedo-Gonzalez JC.

The puncture was executed with a radiofrequency cannula (20G x 100 x 10), an area conspicuously devoid of Teflon. The operator's index finger was introduced into the patient's oral cavity, tracing along the upper jaw. A pivotal directive was to ensure that the needle never exceeded medial limits, safeguarding against inadvertent carotid puncture, as the foramen lacerum resides medially to the upper jaw's edge. The index finger's presence within the oral cavity not only facilitated precise needle guidance, preventing mucosal rupture and oral cavity penetration, but also steered the needle through the space delimited by the ascending branch of the lower jaw and the maxillary tubercle (Figure 1).

With the aid of fluoroscopy imagery, the needle was deftly guided along an imaginary projection line, passing 1 cm anterior to the temporomandibular joint (TMJ), a structural landmark impeccably discernible on strictly lateral radiographs. Upon successful positioning within the foramen ovale, sensory and motor stimulation was meticulously administered to the trigeminal nerve, the gasserian ganglion, and the posterior root. Subsequently, thermorhizotomy was effectuated through the judicious application of C-rF and/or P-rF. The parameters used in C-rF were 90 seconds/60°C, and the P-rF parameters were 60°C for two minutes at 10-second intervals.

Noteworthy modification

A refinement was introduced to the technique, entailing the placement of the foramen ovale within a Cartesian coordinate system denoted by the X, Y, and Z axes [8]. The X coordinate, determined by the upper edge of the maxilla, palpated by the operator's index finger during the procedure, served as a pivotal safeguard against potential carotid vascular complications [8,9]. The Y coordinate was defined by an imaginary point located 1 cm anterior to the TMJ and inferior to the petrous pyramid, all meticulously discerned on strictly lateral radiographs. Meanwhile, the Z coordinate was contingent upon the depth of the needle, guided by the distinct image of the petrous pyramid, its upper edge, and the posterior aspect of the clivus (Figure 1) [13].

Simultaneously, the depth of the needle was meticulously calibrated by the presence of pain at the precise moment of needle contact with the third branch of the trigeminal nerve. The final two centimeters of needle advancement were further guided by sensory and motor stimulation, ensuring a precise and efficacious intervention.

## Results

Following the radiofrequency percutaneous thermorhizotomy procedure, a meticulous evaluation of clinical outcomes ensued, characterized by a discernible reduction in pain intensity as measured on the VAS. This evaluation was scrutinized across three temporal strata: short-term (less than 30 days), medium-term (less than one year), and long-term (greater than one year). In the short term, a substantial amelioration of pain, exceeding 50%, was evident in most patients (94%, 120 patients), demonstrating strikingly similar results in both Group 1 (98%, 74 patients) and Group 2 (90%, 47 patients). The average VAS score was noted at 3/10 for Group 1 and 2/10 for Group 2. In the medium term, notable improvement surpassing 50% in pain intensity was observed in 89% of patients (114), with Group 1 registering 92% (70 patients) and Group 2 at 86% (44 patients). The mean VAS score for this period was recorded at 3.5/10, with Group 2 exhibiting a slightly higher score of 4/10 in contrast to the 3/10 noted in Group 1.

**Figure 2:**
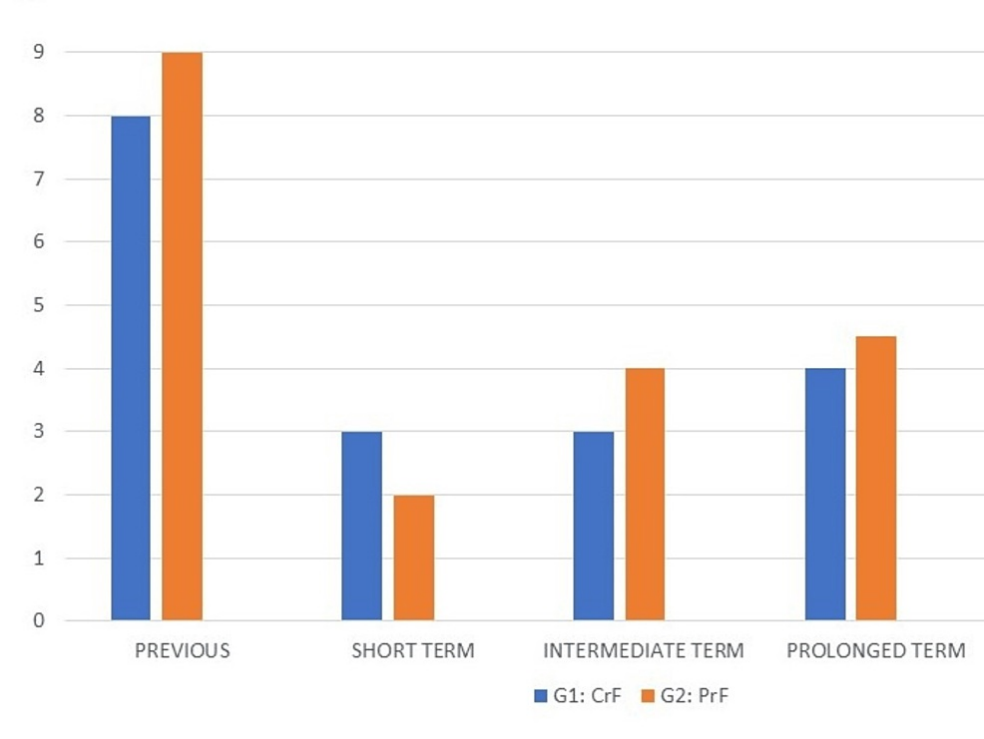
Evolution of pain with treatment based on the visual analog scale (VAS) VAS is shown before the procedure as well as in the short-term, medium-term, and long-term after the procedure. G1: CrF, Group 1: Continuous radiofrequency; G2: PrF, Group 2: Pulsed radiofrequency.

**Figure 3:**
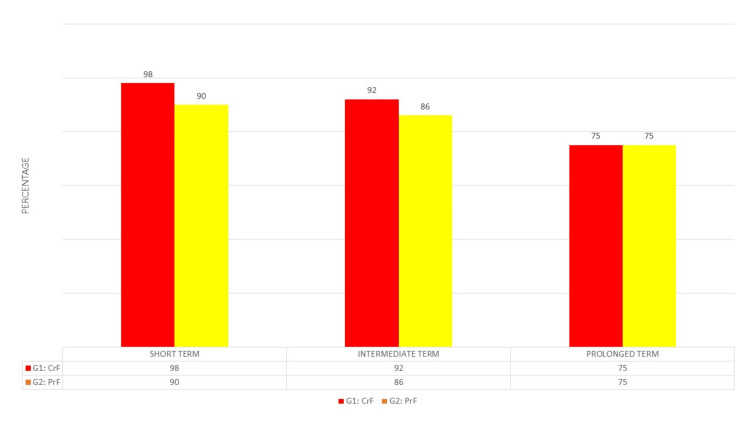
Percentage of patients with improvement of more than 50% in pain G1: CrF, Group 1: Continuous radiofrequency; G2: PrF, Group 2: Pulsed radiofrequency.

In the most recent follow-up, a comparable advancement, exceeding 50% in pain relief, was documented in 75% of patients (96) across both groups, denoting no substantive discrepancies. Medication usage witnessed a discernible reduction, with most patients transitioning to lower doses of antineuropathic agents to mitigate sensory disturbances, occasionally supplemented with an opioid. Among the patient cohort, 18 individuals (14%) necessitated a subsequent percutaneous procedure, with 10 cases (13%) in Group 1 and eight cases (15%) in Group 2. Additionally, microvascular decompression was deemed necessary for six patients (4.6%), evenly distributed across both groups, and radiosurgery was employed as a requisite intervention for three patients (3.9%) in Group 1. Gratifyingly, no instances of mortality were recorded among the patient cohort. However, one patient endured prolonged persistence of a III cranial nerve palsy, exceeding a duration of 30 days. Furthermore, corneal hypoesthesia was observed in eight patients (6.2%), all of whom belonged to Group 1. Facial hypoesthesia was a ubiquitous occurrence in all patients from Group 1 and was additionally noted in six patients (11.5%) from Group 2. A singular patient experienced persistent neuropathic pain necessitating intervention through radiosurgery (Figures 2, 3).

## Discussion

In the early twentieth century, the inaugural forays into percutaneous chemical neurolysis treatments of the branches of the trigeminal nerve were made. Abadie and Verger, in 1901, applied subcutaneous cocaine to the afflicted regions experiencing pain [3]. Following suit in 1903, Schlosser, a German "oculist," employed an intraoral approach via the soft palate to access the foramen ovale and administer an 8% alcohol solution [3]. These techniques traversing the foramen ovale found application in Europe through the efforts of Taptas in 1906 and Harris in 1910, who adopted a lateral approach to reach the foramen ovale and instill alcohol into the gasserian ganglion [3]. Harris's patient found respite from pain and lived an additional 27 years devoid of symptoms [3]. In 1912, Harris published his inaugural series encompassing 1,433 patients treated with this method, encompassing various forms of facial pain alongside headaches [14]. The year 1913 witnessed Hartel's seminal article in a German publication, wherein he introduced his "Hartel pathway" for approaching the gasserian ganglion through the foramen ovale [15]. Through this avenue, alcohol, glycerol (Håkanson, 1981, Karolinska Hospital Stockholm), and mechanical compression employing a balloon (Shelden et al., 1950, and later Mullan and Lichtor, 1979) were applied [16-18]. Kirschner's 1930 publication showcased the utilization of electrosurgery on the trigeminal nerve for pain management [19]. Since 1962, the application of electric current through the foramen ovale, in contact with the gasserian ganglion, found increasing use. Thiry, in 1962, was the pioneer in deploying low-intensity current. The inception of utilizing radiofrequency waves for TN treatment can be traced back to 1969 with Sweet's pioneering work [20]. These radiofrequency waves had earlier applications in other pathologies, first explored by Cushing in the early twentieth century and later by Schurmann in 1972 [7]. The curved electrodes presently in use were conceptualized by Tew and Keller [21]. The theoretical framework for radiofrequency's application was posited by Letcher and Goldring, suggesting that A-delta and C fibers exhibit greater susceptibility to heat compared to A-alpha and beta fibers. This concept allows for selective lesioning of the former at controlled low temperatures [22].

The term "radiofrequency," more commonly recognized as the "radiofrequency spectrum," denotes the segment of the expansive electromagnetic spectrum with the least energy, spanning from 3 kHz to 300 GHz. The historical evolution of radiofrequency finds its origins in the utilization of electricity as a tool for pain management during the eighteenth century [23]. Medicine bore witness to the Second Industrial Revolution, wherein electricity permeated everyday life. Heinrich Rudolf Hertz, a German engineer and physicist, achieved the transmission of radio waves and magnetic waves between two receivers. He dedicated a significant portion of his life to studying electromagnetic waves, formulated the telegraph's fundamental principle, and coined the term "Hertzian waves" for electromagnetic waves, with one Hertz representing one cycle per second. Nikola Tesla, the Serbian engineer, among his myriad achievements, constructed the inaugural radio transmitter enabling the conduction of electromagnetic energy without cables. Much like his construction of the inaugural hydroelectric power station at Niagara Falls, which brought electricity to the city of Buffalo (NYC), Tesla also devised the initial electrotherapy apparatus [23].

In the domain of medicine, a pivotal moment emerged in 1780, when Luigi Galvini employed electricity in the study of animal muscles, discerning their responses and unraveling the intricate connection between their application on the spinal cord and resultant limb contractions [23]. Building on this foundation, luminaries such as Beaunis in 1863 and Fournie in 1873 ventured to apply electricity directly to the cerebral surface, pioneering the development of bipolar stimulation systems. Golsinger, in 1895, achieved a breakthrough by effecting profound lesions within the deep recesses of the brain through monopolar stimulation [2,3]. It was in 1906 that the German physician Karl Franz Nagelschmidt coined the term "Diathermy," denoting the generation of heat within bodily tissues through the agency of a high-frequency electrical current coursing between two electrodes strategically positioned on the skin [5]. From this ingenious concept emerged the diathermic scalpel, wherein one of the electrodes assumes the form of a surgical blade, while the other is carefully placed on the skin's surface. This epochal development garnered fervent advocacy from Cushing, who not only championed the use of diathermy but also advanced the lexicon of "electrosurgery" to encapsulate procedures employing radiofrequency waves for the dual purpose of tissue coagulation and precise sectioning [7]. In the pursuit of pain management, Dansorval, in 1891, ushered in a new era by subjecting human tissues to these waves, substantiating their capacity to elevate tissue temperature [8]. A watershed moment arrived in 1950 with the advent of the first commercially available apparatus for employing radiofrequency in the arena of pain management, an innovation jointly realized by Cosman and Aranow [8]. Some of the benefits of radiofrequency are shown in Table 3.

**Table 3:** Benefits of using radiofrequency for the treatment of trigeminal neuralgia

Benefits of using radiofrequency for the treatment of trigeminal neuralgia
Controlled tissue injury.
Controlled temperature.
Needle position can be verified with stimulation.
The procedure is done under sedation.
Low morbidity.
Quick recovery.
The procedure can be repeated.
The impedance measurement allows us to know the type of tissue that is in contact with the electrode.
The voltage is established between the active electrode and the plate.
The friction produced by the ions in the tissue is what generates heat.
The temperature is the same at the tip of the needle and in the tissue.
The greater the vascularization of the tissue, the longer the exposure time to the wave is needed.

In 1965, Sweet and Wepsic pioneered the application of radiofrequency for addressing lower back pain, marking a significant milestone in the management of various painful pathologies [20]. Subsequently, in 1970, Schaerer documented the inaugural series of treated patients afflicted with facet disease [24]. Then, in 1995, Ayrapetyan et al. introduced the concept of pulsed radiofrequency, also known as isothermal radiofrequency, heralding a novel non-invasive, non-destructive therapeutic approach [11]. This innovative application of radiofrequency engenders electromagnetic fields with profound physiological effects on the cell membrane, inducing structural modifications. Notably, it induces heat at exceedingly low, non-deleterious temperatures, interfacing with tissue through meticulously orchestrated wave cycles that afford the tissue a respite from temperature escalation between cycles. These groundbreaking insights were further honed and refined by the contributions of Cosman and Erdine et al. [9,10].

TN stands as one of the most acute forms of neuropathic pain, a condition historically characterized by evocative terms such as "oris torture," "suicidal nevralgie," and "tic douloureux." Its therapeutic regimen encompasses a multimodal approach involving antineuropathic agents, opioids, and drugs that augment endogenous inhibitory pathways [4,22]. Carbamazepine continues to reign as the antineuropathic of choice, distinguished by its superior efficacy, surpassing even those targeting the alpha 2 delta subunit of calcium channels. Nonetheless, the efficacy of these treatments may wane, or they may be poorly tolerated, thereby perpetuating a decline in the quality of life and enduring excruciating pain. Peripheral anesthetic interventions have shown limited efficacy, and peripheral neuromodulation techniques remain in the nascent stages of development, lacking robust randomized studies. Hence, the definitive management of essential neuralgia of the trigeminal nerve encompasses either microvascular decompression of the nerve at the cerebellopontine angle or percutaneous radiofrequency thermorhizotomy. The latter, involving a percutaneous procedure targeting the gasserian ganglion and the posterior root of the trigeminal nerve through the foramen ovale with radiofrequency, may emerge as the preferred choice over posterior fossa surgery. This preference arises from its reliance on anesthetic neurosedation and its outpatient nature. Continuous radiofrequency treatment is the favored option in elderly patients, especially those with a medical history precluding nerve decompression surgery, notwithstanding the associated risk of neuropathic pain stemming from deafferentation. Conversely, in younger patients, the outcomes of P-rF, which minimizes this risk, present an intriguing avenue for their treatment, extending even to other manifestations of facial pain, postherpetic neuralgia, or lingering neuropathic pain in patients with benign lesions of Meckel's cavum treated with radiosurgery. It is imperative to underscore that the incidence of deafferentation-induced pain in our series is less than 1% (one patient).

A review of the literature over the past eight years across prominent search engines yields a paucity of studies on this subject. We identified nine pertinent articles, including three meta-analyses incorporating systematic reviews, one comprehensive systematic review, four clinical studies (some of which were randomized), and a recent retrospective investigation [25-29]. Most of these contributions emanate from Asian countries such as China, India, and Japan, with only the retrospective study conducted in the United States [29]. A prevailing objective of these studies remains the delineation of whether the use of P-rF, C-rF, or a combination of both confers superior outcomes. Generally, meta-analyses and systematic reviews converge in suggesting that short-term results exhibit comparability across all three techniques, with prolonged follow-up revealing greater recurrence rates in those exclusive to P-rF. Conversely, instances of hypoesthesia are both less prevalent and of shorter duration in patients subjected to P-rF [26,27]. Some ongoing studies are dedicated to exploring new, safer techniques for addressing the gasser ganglion through the foramen ovale [28]. Techniques employing computed tomography (CT) guidance, employing Cartesian templates, are introduced to enhance precision during needle puncture, yielding parallel results across both groups [29]. Recently, a retrospective study scrutinizing C-rF based on the temperature applied to the gasserian ganglion concluded that lower temperatures yield equivalent clinical outcomes with a reduced incidence of clinical complications [30].

The retrospective and observational analysis of this study, without strict or prospective randomization, makes the analysis of the results limited. However, given the few publications related to the subject and the lack of important series on the use of pulsed radiofrequency, this article takes on greater relevance.

## Conclusions

We maintain the conviction that percutaneous thermorhizotomy procedures employing radiofrequency, whether administered continuously or pulsed, targeting the trigeminal nerve through the modified Hartel pathway, represent a secure and valuable intervention for pain management. The inclusion of P-rF, characterized by its sparing of sensory function post-treatment, unveils a substantial avenue for its application among youthful patients and those grappling with alternative forms of facial pain. This option stands as a less intrusive alternative, facilitating swifter recuperation.
